# Postprandial glycaemic and insulinaemic responses in adults after consumption of dairy desserts and pound cakes containing short-chain fructo-oligosaccharides used to replace sugars

**DOI:** 10.1017/jns.2015.22

**Published:** 2015-10-12

**Authors:** J. M. Lecerf, E. Clerc, A. Jaruga, A. Wagner, F. Respondek

**Affiliations:** 1 Nutrition Department, Institut Pasteur de Lille, Lille, France; 2 Research and Innovation Department, Tereos, Marckolsheim, France

**Keywords:** Fructo-oligosaccharides, Sugar-reduced foods, Postprandial glucose response, AUC_0–120 min_, AUC between 0 and 120 min, C_max_, maximum concentration, mITT, modified intention-to-treat, PP, per protocol, scFOS, short-chain fructo-oligosaccharides

## Abstract

The present studies aimed to evaluate the glycaemic and insulinaemic responses, in healthy adults, to short-chain fructo-oligosaccharides (scFOS) from sucrose used to replace sugars in foods. Two study populations aged 18–50 years were recruited and they consumed dairy desserts or pound cakes containing either standard sugar content or scFOS to replace 30 % of the sugar content. For each study, the two products were tested once under a double-blind and cross-over design with at least 7 d between the two tests. Glucose and insulin were measured using standard methods in blood samples collected with a venous catheter for 120 min during a kinetic test. For the dairy desserts, replacing 30 % of the sugars with scFOS significantly reduced postprandial glycaemic (AUC_0–120 min_; *P* = 0·020) and insulinaemic (AUC_0–120 min_; *P* = 0·003) responses. For the pound cakes, the glycaemic response was not altered (AUC_0–120 min_; *P* =  0·322) while the insulinaemic response tended to be lower (AUC_0–120 min_; *P* = 0·067). This study showed that scFOS can be used to replace sugars with the benefit of lowering the postprandial glycaemic response without increasing the insulinaemic response. The effect might be modulated by other parameters (e.g. fat content) of the food matrices.

As an example of dietary fibres, short-chain fructo-oligosaccharides (scFOS) produced from sucrose by a controlled reaction with the enzyme fructo-furanosiadase are not extensively digested nor absorbed in the small intestine, but as prebiotic fibres they will be selectively fermented in the large intestine, providing potential health benefits for the host^(^^1^^)^. Because they are not digested and also have a sweet taste (30 % *v.* sucrose), scFOS can be used to reduce food sugar content or energy while maintaining the same rheological and sensory attributes^(^^2^^)^. In Europe and elsewhere (e.g. Canada), all fibres are considered to provide 2 kcal (8·4 kJ) of energy per g instead of 4 kcal (17·9 kJ) for digestible carbohydrates^(^^3^^,^^4^^)^. Dietary fibres can also be used to lower the postprandial blood glucose response^(^^5^^)^, which is a beneficial physiological effect for subjects who are intolerant to glucose^(^^6^^,^^7^^)^. However, this effect depends on the type of fibres used (soluble, insoluble, viscous, non-viscous, etc.) and should be verified for each of them^(^^5^^)^.

The effects of scFOS on the blood glucose response in humans were studied in a pilot study involving two subjects who either consumed a single dose of pure dextrose or of pure scFOS. The consumption of scFOS did not induce an increase in blood glucose contrary to the consumption of dextrose^(^^8^^)^. More recently the effects of different mixtures of maltitol and scFOS in dairy desserts on the postprandial blood glucose response were studied as a secondary objective in a randomised, double-blind reference-controlled clinical study with eighteen healthy subjects^(^^9^^)^. Maltitol and scFOS were used in combination to totally replace sugars and scFOS was also tested for partial sugar replacement (−30 %). The control food contained 35 g of dextrose. As the study was not powered to study this outcome measure, there was only a trend towards reduced glucose AUC_0–120 min_ when a dairy dessert was reduced in sugars through the addition of scFOS (11 g).

The primary objective of the two present studies was to evaluate the postprandial glycaemic response to two different types of foods, in healthy adults, standard with reduced (−30 % w/w) sugars (e.g. dextrose) content through replacement with scFOS in comparison with a control food containing a standard amount of sugars. The secondary objective was to assess the effect on the insulinaemic response. Dairy dessert and pound cake (traditional sponge cake made using 1 lb (0·4536 kg) each of the four ingredients: flour, butter, eggs, sugars) were chosen as examples of popular foods eaten by both children and adults and typically providing significant amounts of sugar that is difficult to replace without compromising on taste^(^^10^^)^.

## Methods

### Subjects

Healthy subjects were recruited for these two studies according to the same inclusion criteria: age (18–50 years), BMI (18·0 and 25·0 kg/m^2^), used to eating breakfast, non-smoker, not using medication which could affect nutrient absorption, lipid or carbohydrate metabolism. The subjects were screened according to fasting blood glucose level ≤ 1·1 g/l, fasting blood cholesterol ≤ 6·35 mmol/l, TAG ≤ 1·70 mmol/l, insulin ≤ 20 mU/l (139 pmol/l), HbA1c ≤ 7 %, and no clinically significant abnormality concerning complete blood count, and liver enzymes.

All the subjects provided written informed consent to participate after study procedures had been explained to them. The studies were approved by the ethics committee (CPP Nord-Ouest I, Rouen, France) and were performed in accordance with the guidelines of the International Conference on Harmonisation of Good Clinical Practice and the principles laid down in the current version of the Helsinki Declaration.

The two studies were powered (α 0·05; 0·80 power) on the basis of previously reported postprandial glycaemic response induced by the consumption of FOS and sugar products^(^^9^^)^.

After adjustment for drop-out, twenty-five and thirty-five subjects, respectively, were required in the dairy dessert and pound cake studies.

### Experimental design

These two acute crossover, double-blind placebo-controlled, randomised studies were both performed in a single clinical centre (Institut Pasteur Lille, Lille, France).

For each study, the two different foods (standard = control or sugar-reduced) were administered orally, according to the randomisation list, to the subjects during two successive experimental sessions with at least one washout week between them. During each experimental visit, the subjects arrived at the clinical centre in the morning after a 10-h fast and were subjected to a clinical examination and a medical interview. The last dinner before fasting was a calibrated meal given to all the subjects, providing 677 kcal (2833 kJ) including 12 % of energy from protein, 54 % from carbohydrate and 34 % from fat.

### Products being studied

The studied scFOS were FOS from sucrose (Actilight^®^ 950P; Beghin Meiji), comprising about 37 % 1-kestose (GF2), 53 % nystose (GF3) and 10 % 1F-β-fructofuranosyl nystose (GF4). For the first study, the products were administered orally in a dairy dessert containing, in decreasing order of weight: dextrose, cocoa powder, maltodextrins, modified starch, milk protein, chocolate flavour, carrageenan, and sucralose for sweetness adjustment (Table 1). The ingredients were mixed together and heated in two phases: 65°C for 10 min and 85°C for 15 min. After being packaged in cans, they were sterilised at 121°C for 16 min. For the second study, the products were administered in a pound cake containing, in decreasing order of weight: wheat flour, eggs, sucrose, margarine, water, glucose syrup, wheat starch, baking powder (disodium diphosphate, sodium carbonate) and emulsifying agent (Table 1). After mixing all the ingredients at 30°C, the dough was cooked at 170°C for 30 min.

**Table 1.** Nutritional composition of the dairy dessert and pound cake, by portion

	Dairy dessert (210 g)		Pound cake (100 g)	
	Control	Reduced in sugars	Control	Reduced in sugars
Energy content				
kcal	218·9	193·0	461	443
kJ	915·9	807·5	1929	1854
Carbohydrates (g)	48·1	32·1	58·3	49·2
Sugars*	36·0	25·3†	28·1	19·4‡
Lipids (g)	2·2	3·0	22·1	22·1
Proteins (g)	6·1	8·3	6·4	6·4
Fibres (g)	2·7	14·8	1·0	10·1
scFOS (g)	0·0	11·2	0·0	9·1
scFOS recovery in final food (%)	–	100	–	100

scFOS, short-chain fructo-oligosaccharides.

* ‘Sugars’ means all monosaccharides and disaccharides present in food but excludes polyols.

† Sugars reduced by 30 %.

‡ Sugars reduced by 31 %.

Both products were manufactured externally and labelled in exactly the same way by the person in charge of production. The investigators and subjects could not see any difference in term of food presentation and taste, as sweetness, for example, was adjusted to be equivalent in both products.

### Analysis of short-chain fructo-oligosaccharide content in foods

The food samples were dissolved in pure water at 40°C, homogenised and centrifuged. The supernatant fraction was filtered (0·2 µm) and diluted before injection into the chromatograph. They were then analysed by anion-exchange chromatography (Dionex). The samples were hydrolysed by α-glucosidase and invertase and analysed again. The method has been described by Ouarne *et al*.^(^^11^^)^.

### Evaluation of glycaemic and insulinaemic responses

On the day of the test, a nurse placed a catheter on the subject's arm, started the kinetic test for 120 min, and then took the catheter off. The subjects were instructed to eat the dairy dessert or pound cake within 5 to 10 min, under fasting conditions, with 150 ml of water at the clinical site. The kinetic test consisted of sampling venous blood at T-5 and T-1 min before the subject ate the meal, and then at T15, T30, T45, T60, T90 and T120 min after meal intake.

For the kinetic test, the blood samples were collected in sodium fluoride and potassium oxalate for glucose determination, and serum-separating tubes for insulin. The level of blood glucose was assessed by an enzymic UV test (hexokinase method) (AU480; Beckman Coulter) and commercially available glucose reagents (OSR6121; Beckman Coulter). The blood insulin level was assessed by an immunoradiometric assay (Cisbio Bioassays).

Each subject's compliance was checked by the study coordinator during the sessions (meal intake according to protocol and respect of the sampling time).

### Statistical analyses

The results are presented as means and standard deviations. Variables were assessed for normality of distribution using the Shapiro–Wilk test. If the normality assumption was rejected, a log transformation of the data was performed. The incremental AUC between 0 and 120 min (AUC_0–120 min_) for blood glucose and insulin concentrations was computed following the FAO recommendation^(^^12^^)^.

AUC_0–120 min_ and maximum concentration (C_max_) for glucose and insulin were analysed using a mixed-model ANCOVA with ‘product’ as fixed effect, ‘subject’ as random effect and glucose or insulin baseline value as covariate.

A statistical analysis was conducted on the modified intention-to-treat (mITT) population and on the per protocol (PP) population using SAS^®^ software version 9.1.3 (SAS Institute Inc.). For all the statistical tests, a 0·05 significance level was used to claim a statistically significant effect.

## Results

### Short-chain fructo-oligosaccharide content in foods

Analysis of the foods after cooking and heat treatment showed that 100 % of scFOS was recovered (Table 1) and the ratio between GF2, GF3 and GF4 was identical to that of the ingredients (data not shown).

### Study populations

Two different study populations were recruited to participate in the studies: one to test the dairy dessert and one to test the pound cake.

### Study 1: dairy dessert

**Fig. 1.**
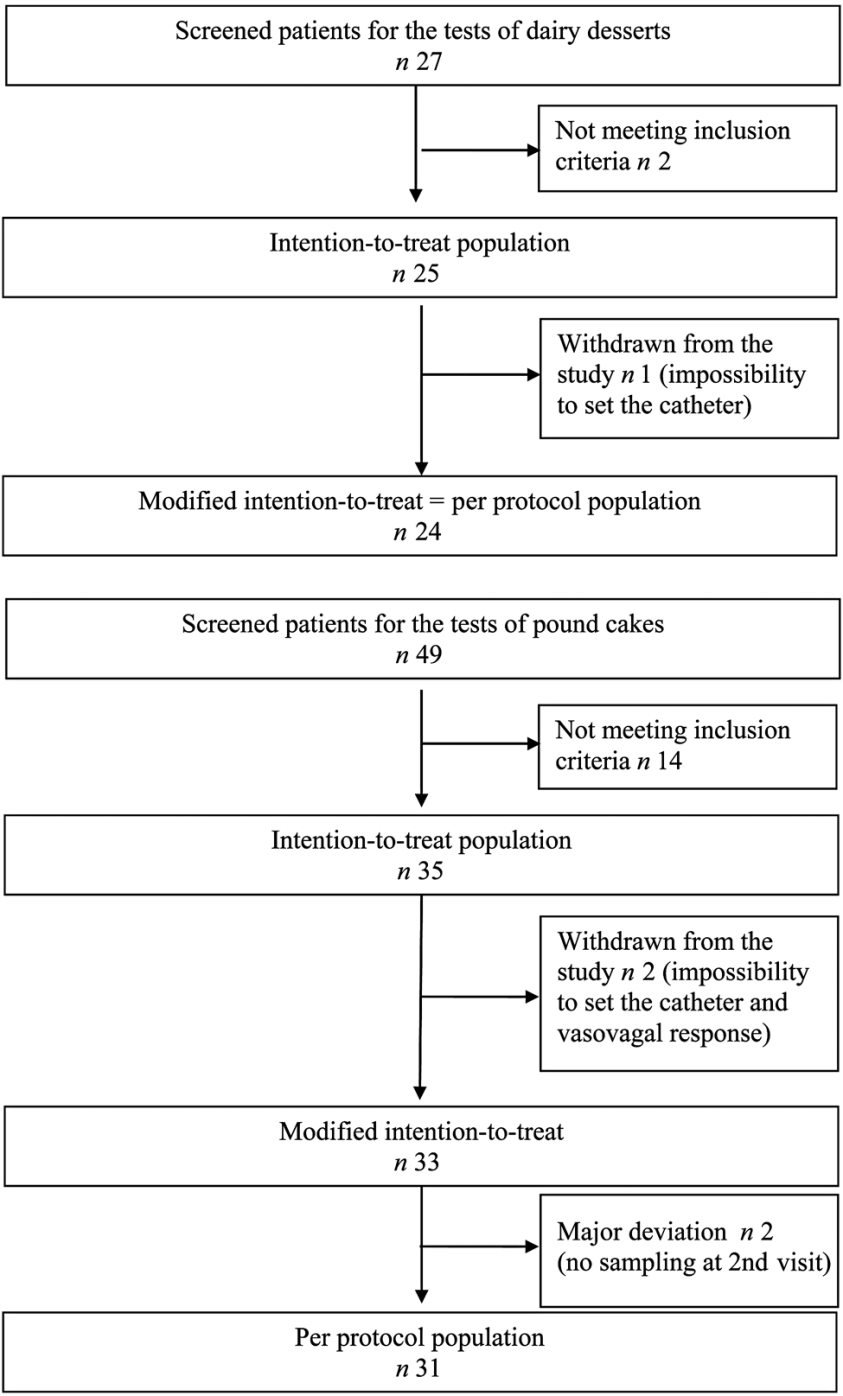
Distribution of subjects among intention-to-treat and per protocol populations.

The mITT and PP populations were equivalent in this study (Fig. 1). This population was 32·3 (sd 8·7) years old on average, with an average BMI of 22·3 (sd 1·9) kg/m^2^. Of the subjects, 28 % were male and 72 % female. The average fasting glycaemia of the ITT population was 4·88 (sd 0·50) mmol/l and insulinaemia was 3·68 (sd 1·52) mU/l (25·56 (sd 10·56) pmol/l), with no significant difference between the two visits.

### Study 2: pound cake

The mITT population represented thirty-three out of the thirty-five randomised subjects and the PP population (results not shown) was composed of thirty-one randomised subjects who completed the study without any major deviation. Two subjects were excluded from the mITT because they left the study prematurely for no specific reason before product consumption, and two subjects were excluded from the PP population because several blood samples used to evaluate the blood glucose response could not be taken during the 2nd visit. The randomised population was 31·9 (sd 8·1) years old on average with an average BMI of 21·9 (sd 1·9) kg/m^2^. Of the subjects, 29 % were male and 71 % female. The average fasting glycaemia of the ITT population was 4·86 (sd 0·37) mmol/l and insulinaemia was 4·27 (sd 1·71) mU/l (29·66 (sd 11·88) pmol/l), with no significant difference between the two visits.

### Glycaemic and insulinaemic responses

**Fig. 2.**
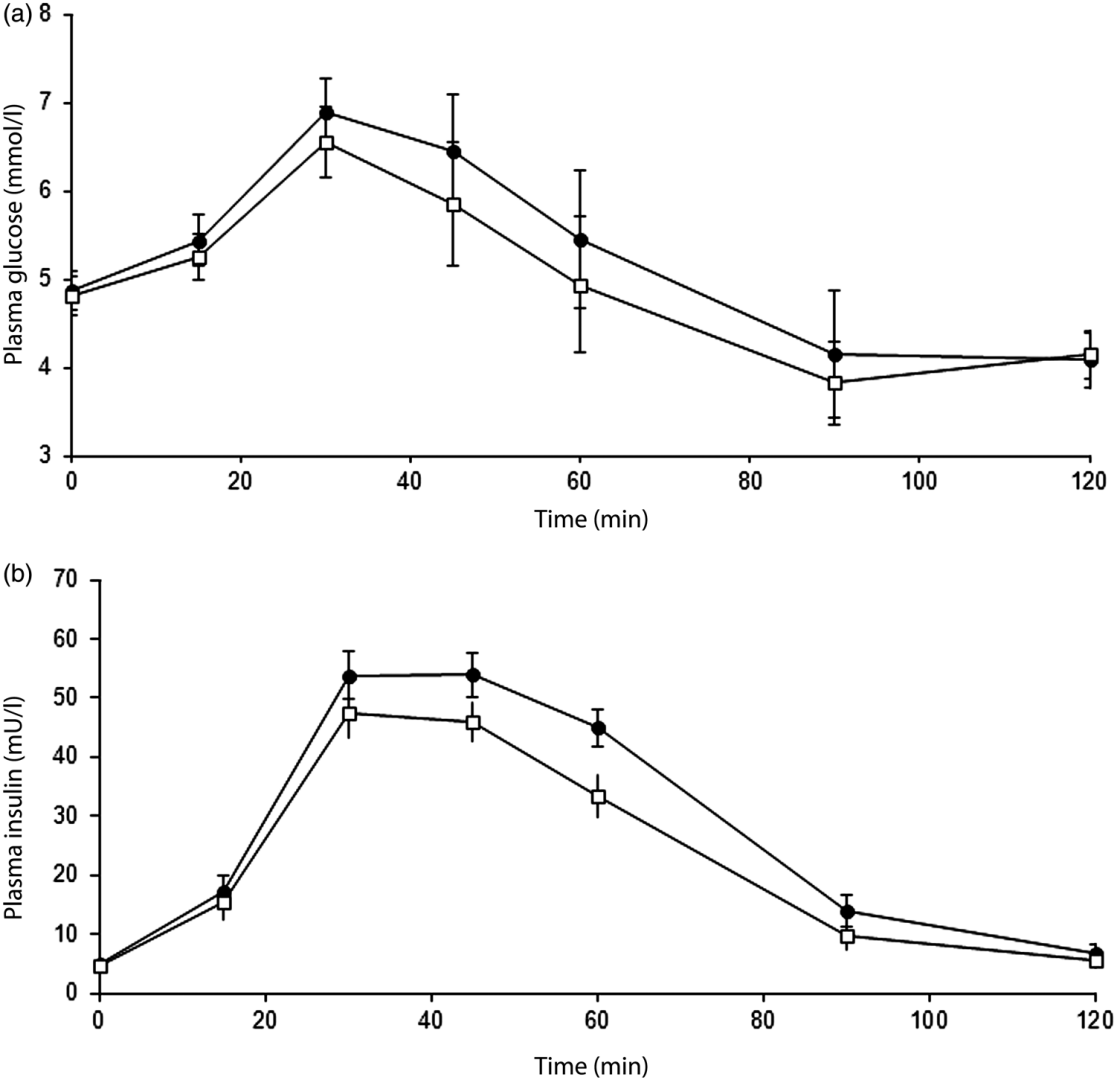
Postprandial (a) plasma glycaemic and (b) plasma insulinaemic responses over 120 min after taking the dairy dessert containing 35 g dextrose (control; –●–) or 24 g dextrose and 11 g short-chain fructo-oligosaccharides (scFOS; –□–) in the modified intention-to-treat population (*n* 24). Data are means, with standard errors represented by vertical bars. Glucose and insulin AUC were significantly lower following scFOS-containing products than following control (*P* = 0·020 and *P* = 0·003, respectively) (mixed-model ANCOVA). To convert insulin in mU/l to pmol/l, multiply by 6·945.

Consumption of the dairy dessert, formulated with scFOS replacing part of dextrose (−30 %, w/w), induced a lower postprandial blood glucose response compared with dextrose in the mITT population whereas the C_max_ was not altered (Table 2, Fig. 2). In parallel, the insulin response as illustrated by the AUC_0–120 min_ was also lower after consumption of the sugar-reduced dairy dessert than with the standard one without impact on the C_max_ (Table 2, Fig. 2).

**Table 2.** AUC_0–120 min_ and maximum concentration (C_max_) of plasma glucose and insulin for 2 h after consumption of dairy dessert or pound cake with standard or reduced sugar content in the modified intention-to-treat population (Mean values and standard deviations)

	Dairy dessert (*n* 24)					Pound cake (*n* 33)				
	Control		Reduced in sugars			Control		Reduced in sugars		
	Mean	sd	Mean	sd	Dessert effect: *P*	Mean	sd	Mean	sd	Cake effect: *P*
Glucose										
AUC (mmol × min/l)	92·8	90·6	69·3	56·0	0·020	69·0	69·8	62·0	55·8	0·340
C_max_ (mmol/l)	7·3	1·4	6·9	1·2	0·184	6·3	0·1	6·1	0·1	0·272
Insulin										
AUC (mU × min/l)*	2867	1190	2232·4	1121	0·003	1885·5	1000	1695·5	847·0	0·082
C_max_ (mU/l)*	64·8	25·4	57·5	25·0	0·122	35·0	16·9	31·6	15·0	0·245

* To convert insulin in mU/l to pmol/l, multiply by 6·945.

**Fig. 3.**
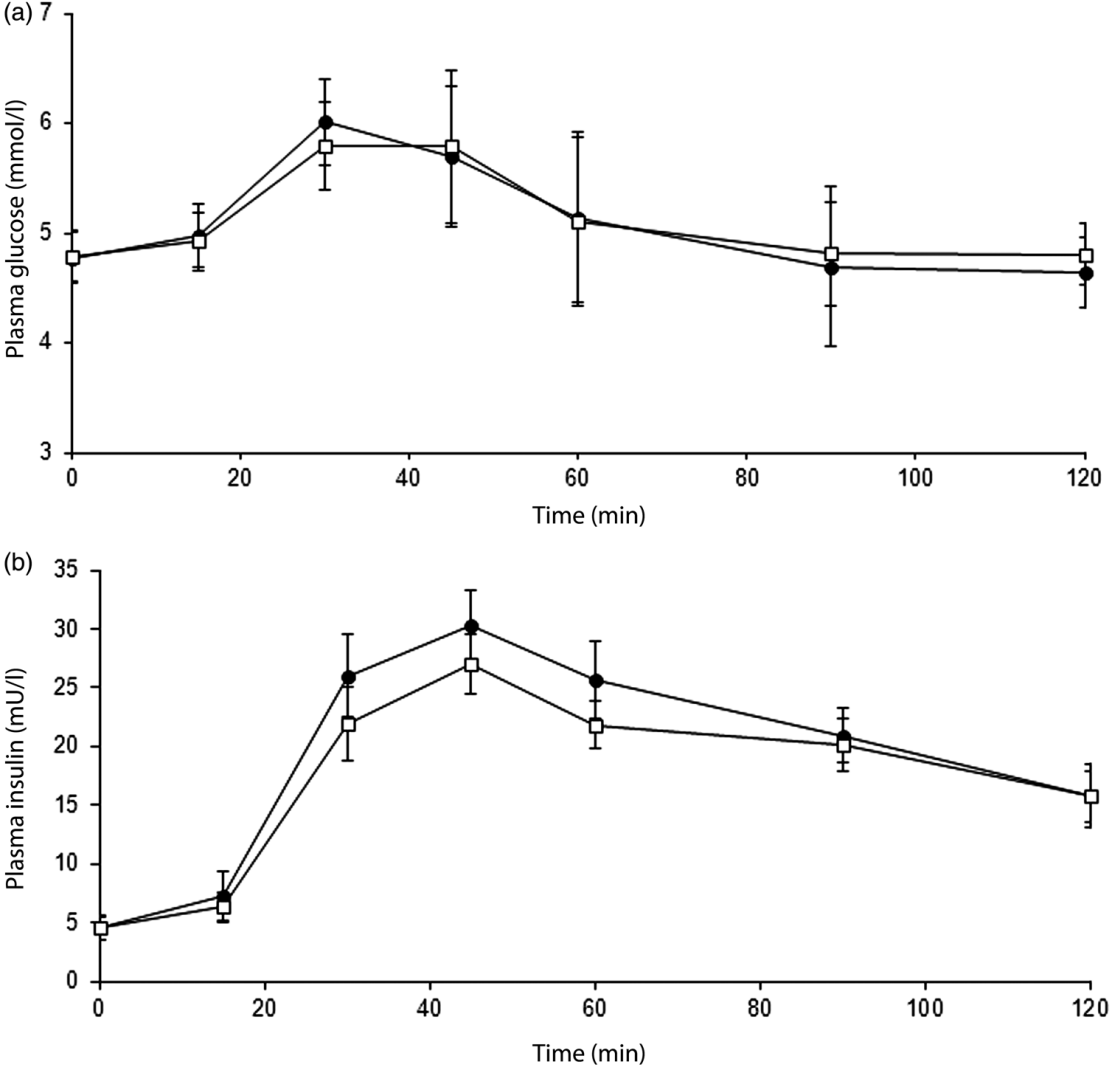
Postprandial (a) plasma glycaemic and (b) plasma insulinaemic responses over 120 min after eating the pound cake containing 28 g sugars (control; –●–) or 19 g sugars and 9 g short-chain fructo-oligosaccharides (–□–) in the modified intention-to-treat population (*n* 33). Data are means, with standard errors represented by vertical bars. To convert insulin in mU/l to pmol/l, multiply by 6·945.

Consumption of the pound cake formulated with scFOS replacing part of the sugars (sucrose and glucose syrup; −31 %, w/w) did not modify the postprandial blood glucose response or the insulin response compared with the standard recipe in the mITT population to any significant extent (Table 2, Fig. 3). Similar results were obtained for the PP population which differed only by two subjects.

### Adverse events

No serious adverse events occurred during the two studies. Six minor adverse events were reported, not linked to the study products but possibly linked with the research procedures (bruising at the blood sampling area). No statistically significant association was made between the ingredients and the imputation of adverse events.

## Discussion

While a reduced postprandial blood glucose response is acknowledged as a beneficial effect, especially for subjects intolerant to glucose, acceptance of sugar-reduced foods is generally less than it is for standard foods^(^^13^^)^ because sugars provide sweetness but also other rheological functions (structure, volume, flavour and aroma, etc.)^(^^10^^,^^14^^)^.

Dietary fibres such as scFOS are sometimes used to partially replace sugars in foods and beverages^(^^2^^)^ because they have a sweet taste and also have a lower energy value than digestible carbohydrates^(^^15^^)^. They have already been successfully used in different types of food reduced in sugars (up to 30 %) in comparison with a control product, without compromise on taste as evaluated by a sensory panel^(^^2^^,^^16^^)^. Furthermore, using scFOS to partially replace sugars also helps enrich foods with fibres, the consumption of which, in Europe, is generally lower than what is recommended^(^^17^^)^.

The present study aimed to evaluate, in healthy adults, the postprandial glycaemic and insulinaemic responses of two types of food matrices with or without scFOS in replacement of sugars. A dairy dessert containing 35 g dextrose was chosen as a first example; the dose of dextrose was partially replaced by 11 g of scFOS in the sugar-reduced recipe. This level of reduction was chosen according to European Union regulation for the claim ‘reduced in sugars’ which requires at least a 30 % (w/w) reduction in comparison with a reference product. The same principle was applied to formulate the pound cake that initially contained 28 g of sugars (sucrose and glucose syrup) for the consumed portion. The test pound cake provided 9 g of scFOS.

All scFOS added to the recipe were recovered after cooking and heat treatment, confirming the stability of scFOS in food matrices. Indeed, it is generally acknowledged that scFOS are stable during heat treatment but may be sensitive to interaction with an acidic pH and high temperature^(^^18^^,^^19^^)^.

The postprandial glycaemic response of the dairy dessert containing the scFOS was reduced in comparison with the glycaemic response of the control dairy dessert, as illustrated by a lower AUC_0–120 min_. This lower glycaemic response was not due to hyperinsulinaemia induced by scFOS because the postprandial insulin AUC_0–120 min_ was also reduced and the insulin spike was not altered compared with the control dessert. This might be explained by the fact that in humans, scFOS are mostly neither digested nor absorbed in the small intestine but completely fermented in the large intestine^(^^15^^,^^20^^)^. Indeed, while pancreatic enzymes cannot hydrolyse scFOS^(^^21^^,^^22^^)^, bifidobacteria and some other bacterial groups possess the β-fructosidase enzyme necessary to hydrolyse the β-(2,1) glycosidic linkages in scFOS as demonstrated *in vitro*^(^^23^^,^^24^^)^. Their fermentation leads to the production of SCFA such as acetate, propionate, butyrate and also CO_2_. Consequently, it was previously shown in two subjects that contrary to dextrose, scFOS consumed as such do not increase postprandial blood glucose or insulin^(^^8^^)^. The present study also confirms a more recent observation that when replacing dextrose by up to30 % (w/w) with scFOS could tend to reduce the postprandial glucose AUC_0–120 min_ of a dairy dessert while not increasing the insulinaemic response^(^^9^^)^.

While being numerically lower with the scFOS, the postprandial glycaemic and insulinaemic responses following consumption of the pound cake reduced in sugars with scFOS did not differ from the responses induced by the control cakes. These different results highlight the fact that factors other than the relative quantities of sugars interact with the postprandial glucose response to foods. This cannot be linked to hydrolysis of scFOS during heating, because they are stable and were completely recovered in the final form of both foods. One hypothesis may be that the quantity of glycaemic carbohydrates compared might be too low. Traditionally for evaluating the glycaemic index, the test load is made with 50 g of available carbohydrates and a recommendation is made to use a test dose not less than 25 g of available carbohydrates^(^^25^^)^. In the present study the quantities of food to be eaten were defined to be as closely as possible representative of reasonable consumption of the considered food. The portion of pound cake contained 28 and 19 g of sugars, respectively, for the control and the sugar-reduced versions. These quantities may not be large enough to observe a significant difference in glycaemic response when these sugars are consumed within a complex food matrix and not as single ingredients.

The two food matrices that were tested also differed in consistency and fat content and these parameters could influence the glycaemic response of food. Studies indicate than a more compact and viscous meal (like the pound cakes in our study) tends to delay gastric emptying^(^^26^^,^^27^^)^. Since the pound cake can be considered as a solid meal while the dairy dessert is more a semi-solid meal, this could explain why blood glucose concentration did not behave the same way with the two matrices. While the dairy dessert was also very low in fat (less than 1·5 %), the pound cake contained around 22 % of fat. Fat is well known to slow down gastric emptying and thus could indirectly make an impact on the arrival of glucose in the bloodstream^(^^28^^–^^31^^)^. Interestingly, the time to the glucose spike in our study was delayed by about 10 min for the pound cake compared with the dairy dessert.

This study highlights the fact that, when used in place of sugars (w/w) for partial replacement, scFOS may help reduce the postprandial glycaemic and insulinaemic responses to foods as illustrated by the dairy dessert here. This is certainly explained by the fact that scFOS are non-digestible carbohydrates and that they are used to replace sugars which are fully available, without making an impact on the palatability of the tested foods^(^^2^^,^^32^^)^. Longer-term studies or second meal effect studies would be of interest in order to see whether scFOS could have other effects on the regulation of blood glycaemia and insulinaemia via their modulation of the activities of gut microbiota as has already been observed in an animal model harbouring human microbiota^(^^33^^)^.
